# How to quantitatively evaluate safety of driver behavior upon accident? A biomechanical methodology

**DOI:** 10.1371/journal.pone.0189455

**Published:** 2017-12-14

**Authors:** Wen Zhang, Jieer Cao, Jun Xu

**Affiliations:** 1 Department of Automotive Engineering, School of Transportation Science and Engineering, Beihang University, Beijing, China; 2 Advanced Vehicle Research Center, Beihang University, Beijing, China; 3 Shenyuan Honors College, Beihang University, Beijing, China; 4 Department of Applied Mechanics, Chalmers University of Technology, Gothenburg, Sweden; Chongqing University, CHINA

## Abstract

How to evaluate driver spontaneous reactions in various collision patterns in a quantitative way is one of the most important topics in vehicle safety. Firstly, this paper constructs representative numerical crash scenarios described by impact velocity, impact angle and contact position based on finite element (FE) computation platform. Secondly, a driver cabin model is extracted and described in the well validated multi-rigid body (MB) model to compute the value of weighted injury criterion to quantitatively assess drivers’ overall injury under certain circumstances. Furthermore, based on the coupling of FE and MB, parametric studies on various crash scenarios are conducted. It is revealed that the WIC (Weighted Injury Criteria) value variation law under high impact velocities is quite distinct comparing with the one in low impact velocities. In addition, the coupling effect can be elucidated by the fact that the difference of WIC value among three impact velocities under smaller impact angles tends to be distinctly higher than that under larger impact angles. Meanwhile, high impact velocity also increases the sensitivity of WIC under different collision positions and impact angles. Results may provide a new methodology to quantitatively evaluate driving behaviors and serve as a significant guiding step towards collision avoidance for autonomous driving vehicles.

## 1. Introduction

Critical vehicle crash conflict (CVCC) situation refers to a scenario within the final seconds and milliseconds before actual collision. Drivers’ reactions upon critical crash conflict right before the accident may determine the severity of accident consequences in a large extent [1–3]. Previous researches have revealed that both extrinsic factors, e.g. drugs [4, 5], alcohol [4, 6–8], weather [9, 10], light conditions [10], scenario’s kinematics [11], as well as intrinsic factors, e.g. personality [12, 13], age [6, 14], experience [4, 7, 15], visual performance [16], cognition type [17–20] may jointly contribute to the final driver response manners. Therefore, a series of experiment designs, carryout, analyses and simulations focus on the effect of those factors on driving behaviors subjected to critical situations by creating simplified scenarios using driving simulators [21–23]. To gain a more realistic driving behavior upon CVCC situation, realistic vehicles were adopted with the installation of data collector, providing further data analysis [24, 25]. Also, driver models based on experiments have been developed to analyze pre-accident scenarios [26]. However, little work uses direct biomechanical indicators, rather than oversimplified indices, e.g. impact velocity, angle, to quantitatively evaluate the effectiveness of critical response.

On the other hand, different CVCC situations may result in various driving behaviors with different response mechanisms, although drivers, especially for inexperienced ones, usually behave based on their intuitions or random choice of swerve to the road sides [27]. For example, in a frontal impact accident, the typical responses of drivers are straightening their arms and bracing backward to seats [28]; in a rear-end collision, drivers tend to brake more quickly with an increasing situational urgency [29]. No systematic study about driving behavior upon various CVCC situations is available since it is rather difficult to control crash angle, speed and timing simultaneously using driving simulators, not to mention in real-world vehicles.

To quantitatively establish the relationship between driving behavior and accident consequence upon CVCC situation is still lacking with limited studying samples and time-consuming procedures. Additionally, collision avoidance (CA) technology is a crucial subset of active safety, which is generally applied to avoid a potential collision in autonomous driving system [30]. Various algorithms for evaluating collision scenarios of autonomous driving were investigated in previous literatures using data from both real traffic situations and simulated scenarios, including threat-assessment algorithm and multilevel collision mitigation method [30–32], while recent fast development of autonomous driving requires algorithms equipped with more rational driving behavior reactions upon possible evitable accidents. On the other hand, numerical simulation may provide a possible solution with much improved efficiency and sufficient data to cover all possible scenarios. To bridge this gap, in this study, a novel methodology of drivers’ spontaneous reaction evaluation based on numerical simulation with validate models is proposed. In Section 2, the methodology would be described in detail and all model related information including accident scenario setting and the biomechanical indicator is depicted. In Section 3, a typical result would be presented and analyzed, underlying the mechanisms between driving behavior and passenger injury outcome. In addition, assessment of drivers’ spontaneous reaction is provided. A further detailed discussion with coupling effects of various impact scenarios is considered.

## 2. Methods

**Fig 1:**
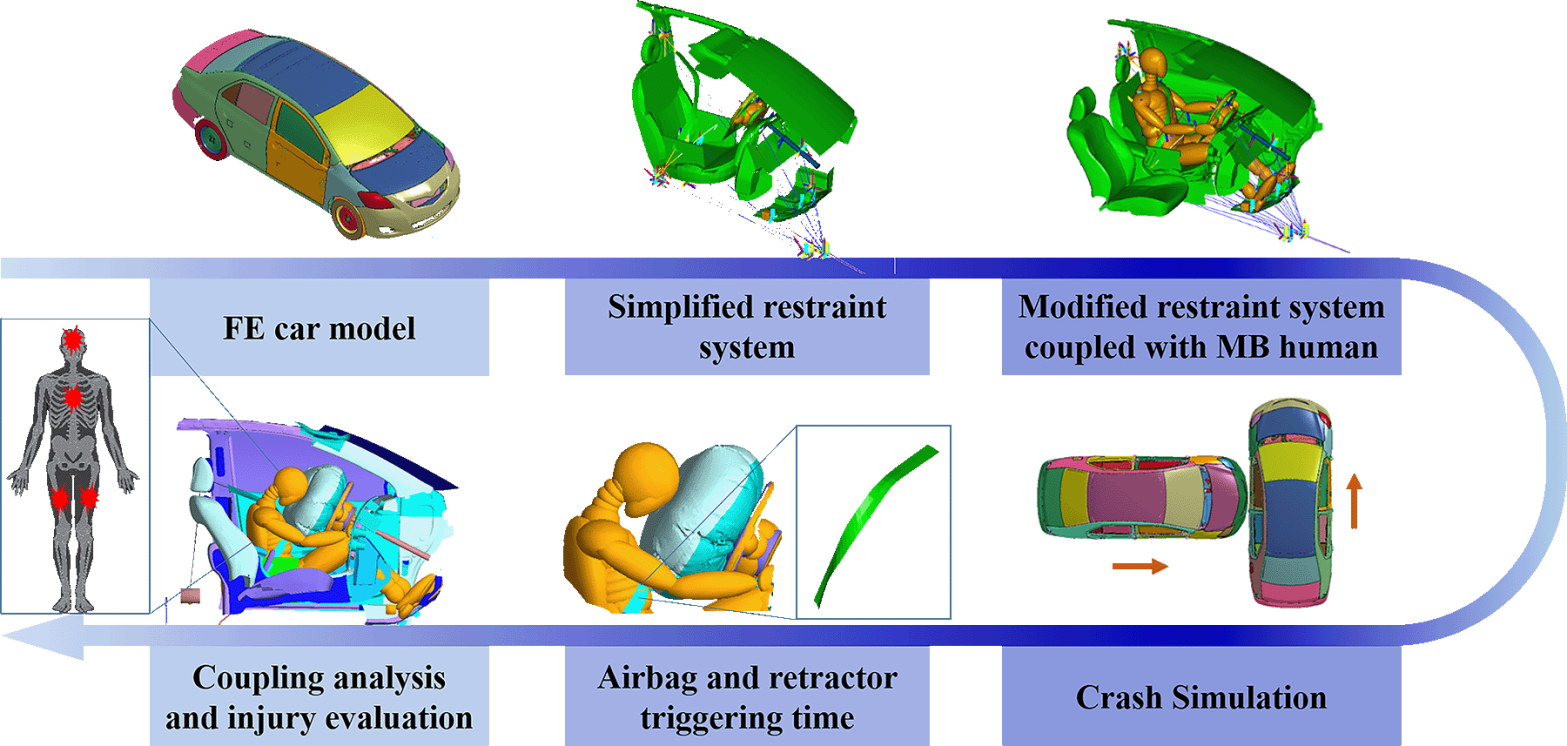
Schematic flow-chart of the proposed methodology by using coupled FE and MB models.

To improve the computation efficiency, FE couple with MB computation strategy is suggested in Fig 1 without the loss of accuracy. Vehicles are depicted by full FE way to obtain a complete and accurate crash pulse while the detailed passenger cabin is described by MB with fully validated passenger dummy model [33].

### 2.1 Traffic accident scenarios

To evaluate drivers’ spontaneous reactions in traffic accidents, a series of traffic accident scenarios in view of impact velocities, angles and positions at the cross road are set. It is worth noting that only side impact cases are taken into account to demonstrate the methodology in this work.

**Fig 2:**
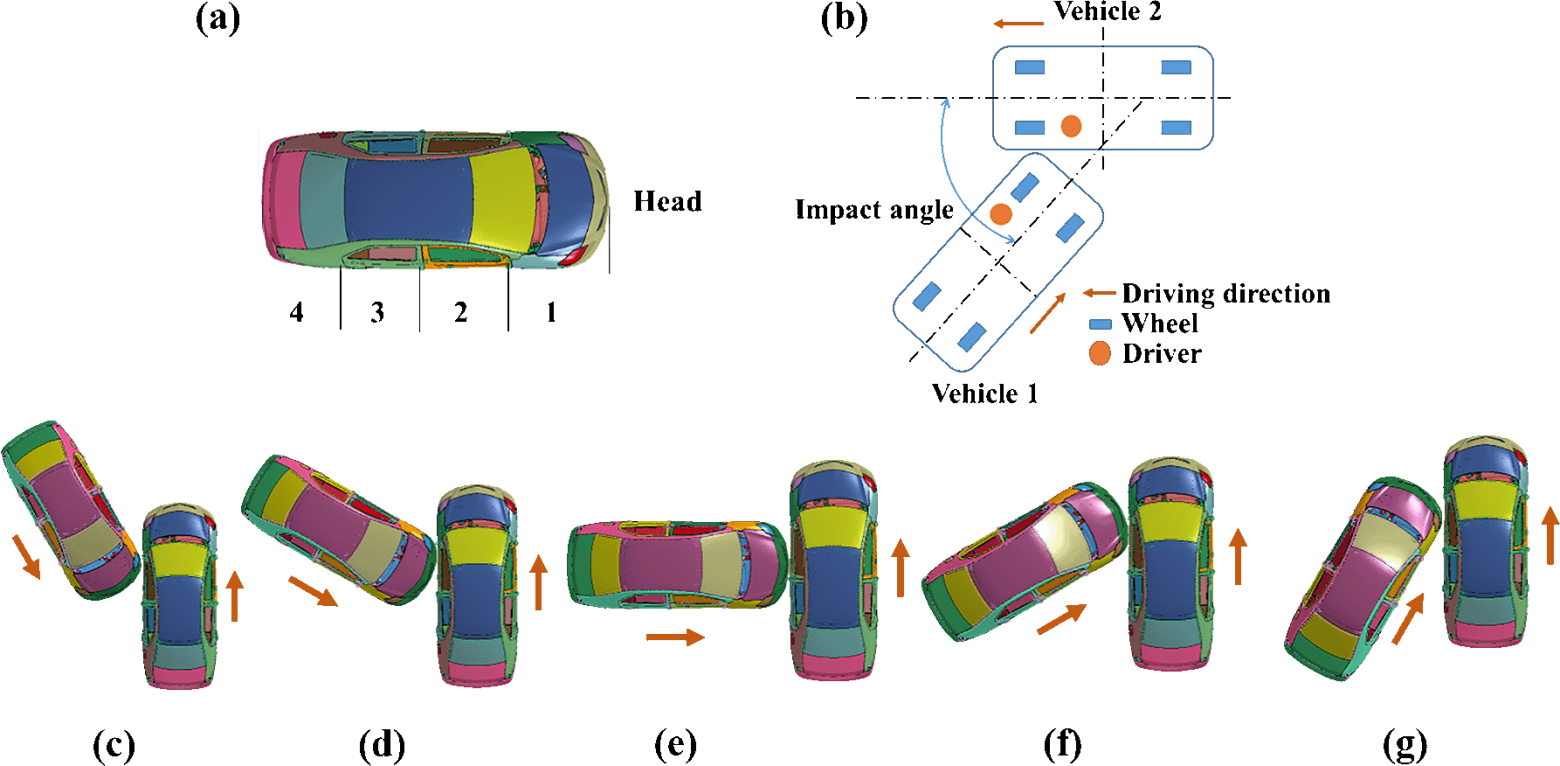
Impact scenario parameters with (a) four impact positions, (b) schematic diagram of impact angle in CVCC situation, impact angles of numerical simulations are (c) 30^o^, (d) 60^o^, (e) 90^o^, (f) 120^o^, (g) 150^o^, respectively.

The impact velocity is far more complicated due to the various pre-crash maneuvers and crash situations. To cover the wide impact speed range, three different impact velocities, i.e. 25 mph, 35 mph, and 45 mph were selected as input conditions to create crash scenarios. To further quantify the collision points on vehicles, we divide the impact positions into four parts (position 1, position 2, position 3 and position 4) evenly along the longitudinal direction of vehicle demonstrated in Fig 2A. In the meantime, five impact angles ranging from 30^o^ to 150^o^ (30^o^, 60^o^, 90^o^, 120^o^, 150^o^) are selected, defined in Fig 2B to 2G.

The parametric matrix of various traffic accident scenarios is listed in Table 1. In this case, we have 60 crash scenarios and in essence, representing various outcomes of driver’s spontaneous reaction upon CVCC situations. Consequently, there are altogether 60 simulation cases and it takes about 80 hours with eight cores in LS-DYNA and about 2.5 hours with two cores in MADYMO for each case.

**Table 1:** Parametric matrix of traffic accident scenarios.

No.	Position	Velocity(mph)	Angle
**1**	Position 1	25	30^o^
**2**		35	60^o^
**3**		45	90^o^
**4**			120^o^
**5**			150^o^
**6**	Position 2	25	30^o^
**7**		35	60^o^
**8**		45	90^o^
**9**			120^o^
**10**			150^o^
**11**	Position 3	25	30^o^
**12**		35	60^o^
**13**		45	90^o^
**14**			120^o^
**15**			150^o^
**16**	Position 4	25	30^o^
**17**		35	60^o^
**18**		45	90^o^
**19**			120^o^
**20**			150^o^

### 2.2 FE vehicle model

**Fig 3:**
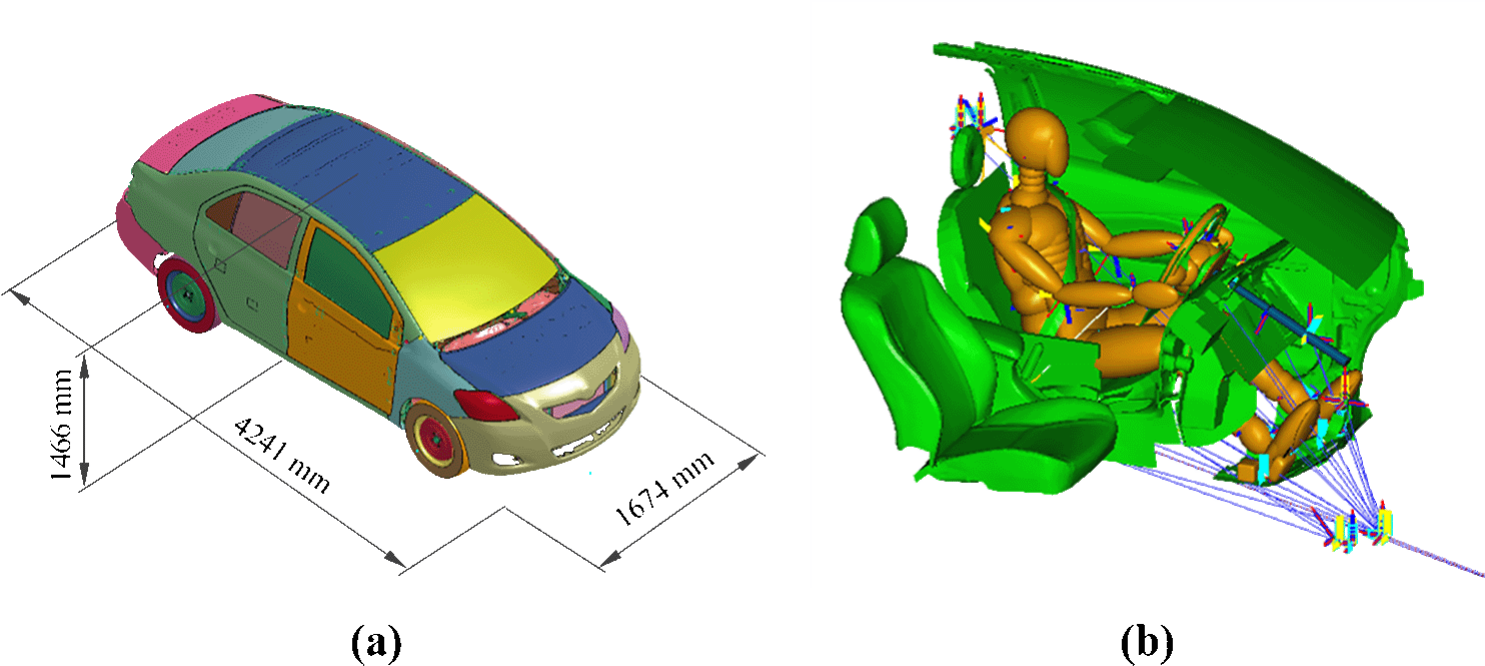
(a) The rough profile and the corresponding dimensions of 2010 edition aof Toyota Yaris sedan finite element model; (b) The Hybrid III 50th percentile dummy model used in the present study and occupant restraint system model provided by NCAC.

The FE vehicle model used in this study is 2010 edition of Toyota Yaris sedan model developed and validated by National Crash Analysis Center (NCAC) under a contract with National Highway Traffic Safety Administration (NHTSA) of the US DOT. Fig 3A illustrates the profile and corresponding dimensions of vehicle model. More information about this model is available on NCAC website [34]. To simplify and eliminate possible crash incompatibility problems, a same FE vehicle model is used. The vehicle-to-vehicle friction coefficient is set to 0.2 according to previous literature which analyzed the frontal vehicle-to-vehicle crash scenarios [35]. The vehicle-to-ground friction coefficient is set to 0.8 to mimic real-world general impact process with braking.

### 2.3 Human model with restraint system

As shown in Fig 3B, the fully validated and widely accepted by automotive industry as human injury evaluation index, Hybrid III 50th percentile Dummy model is chosen for this study for its valuable application in injury analysis [36–38]. This model is given as an include file “d_hyb350el_inc.xml”, consisting of rigid elliptical structures. More detailed information about this human model is presented in Ref.[39].

To mimic the real-world setup for drivers, occupant restraint system model for impact scenarios should be built. Major parts of the restraint system in available FE model constructed and validated by NCAC for frontal impact scenarios are converted to MADYMO platform, also shown in Fig 3B. In the meantime, “7–30” rule suggested by NCAC [40] is applied to set airbag and retractor triggering time. It is worth noting that the retractor triggering time is set to be 15 ms ahead of the airbag triggering time for successful synergic efforts between airbag and retractor.

Appropriate contact types are crucial to gain satisfactory results. In accordance with the model settings by NCAC, user-slave contact is chosen as the type of vehicle interiors-human arms contact, vehicle interiors-human body contact, seats-human body contact and door-human body contact in the present study. In addition, a friction coefficient of 0.3 is used for seats-human body contact and 0.1 is used for other three contacts. The vehicle interiors-human’s lower limbs contact is set as the user-master type and the friction coefficient is 0.5. The input data of this model is the crash pulse obtained from initiative vehicle (vehicle 1) in various simulated vehicle-to-vehicle crash scenarios mentioned before.

### 2.4 Injury evaluation index

Injury indices serve as quantitative measurements to the consequences of drivers’ spontaneous behaviors, i.e. various impact scenarios. Occupant injury during vehicle accidents may involve head, neck, chest, femur or other body parts [41–43]. As such, for a fair comparison, Weighted Injury Criterion (WIC), a multi-criteria analysis combined with head, thorax and femur injury, widely used for quantitatively evaluating driver’s general injury is adopted. WIC can be expressed as [44].

$$\mathrm{WIC}=0.6\left(\frac{\mathrm{HIC}}{1000}\right)+0.35\left(\frac{C_{3\mathrm{ms}}}{60}+\frac{D}{75}\right)/2+0.05\left(\frac{F_{FL}+F_{FR}}{20.0}\right)$$ (1)

where the head injury criteria (HIC) measured over 36 ms is used to evaluate the head injury of the dummy model [45], *C*_3ms_ and *D* are 3-ms chest acceleration and chest deflection, respectively, which can assess the thorax injury of the dummy model during traffic accidents. Left and right femur loads (*F*_*FL*_ and *F*_*FR*_) are also considered in WIC to take femur injury into account. Values of all variables defined above could be numerically calculated based on numerical computation results.

## 3. Results

**Fig 4:**
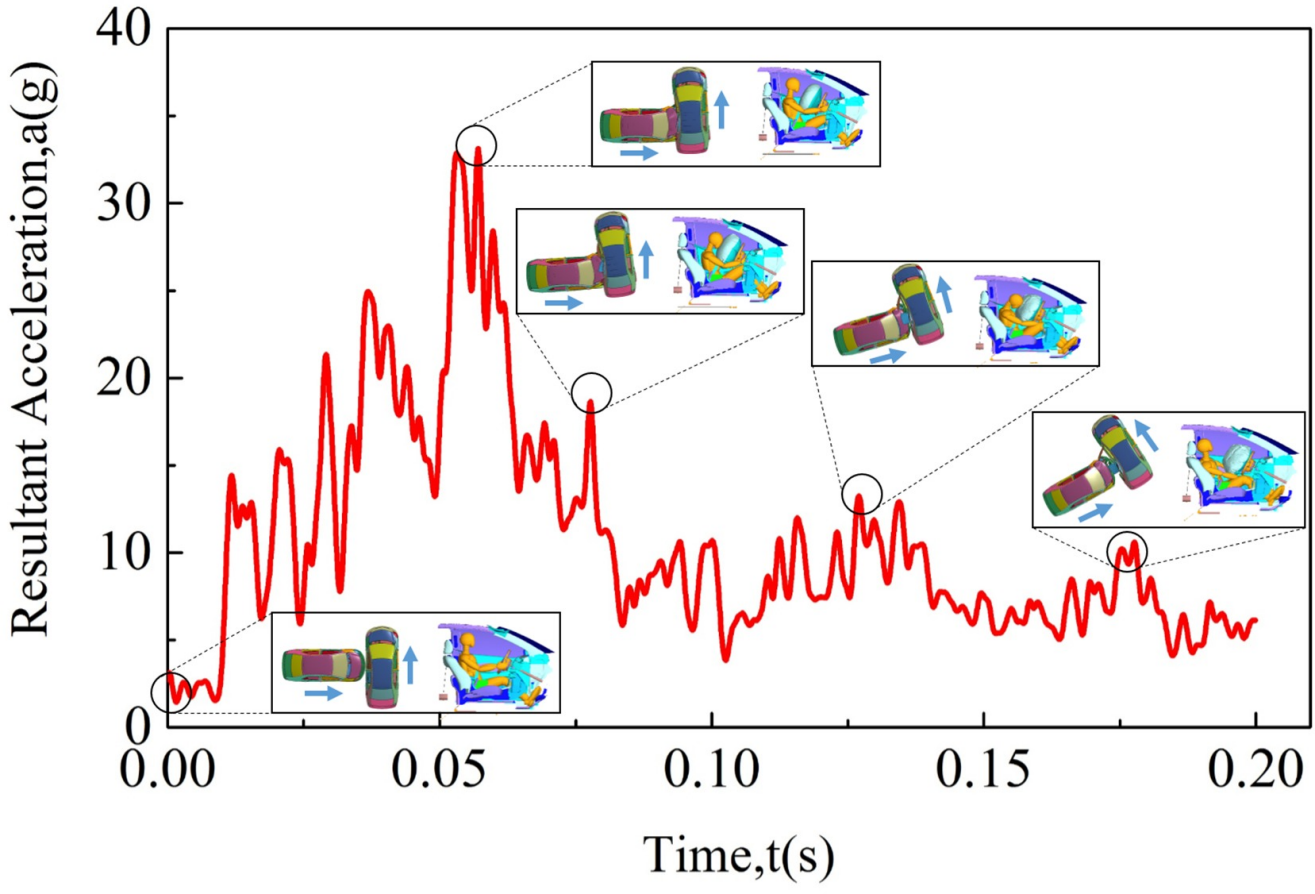
The typical resultant acceleration curve with an impact angle of 90^o^, impact velocity of 35 mph and collision position of position 2, combined with the deformation animation of vehicles and the corresponding response of occupant restraint system model.

Crash pulse is obtained via FE simulation of two identical vehicles modeled above. As shown in Fig 4, resultant acceleration curve under velocity of 35 mph, impact angle of 90^o^ in impact position 2 is chosen to illustrate the typical curve.

Intuitively, one may observe a distinct peak of about 33 g during the crash. In the initial stage, the interaction between two vehicles with initial velocities plays a leading role in the up-climbing phase of resultant acceleration-time curve where the kinetic energy decreases sharply. In accordance with the deformation patterns of two vehicles, energy absorption structures, such as, bumper, crash box, etc. may be essential to protect passenger cabin during this stage.

Shortly afterwards, the resultant acceleration reaches the maximum value when the kinetic energy is changing most drastically when the interaction among high stiffness vehicle parts takes effect.

After the strong collision, two vehicles run apart, reducing the resultant acceleration and the rate of deformation of vehicle parts is also reduced. It is obvious that the front part of initiative vehicle (vehicle 1) and the side part of passive vehicle (vehicle 2) experience quite extensive damage. In the meantime, the corresponding response of occupant restraint system model at different stages is also exhibited in Fig 4, where the airbag fully deploys at the distinct peak of resultant acceleration curve.

During the declining stage of resultant acceleration curve, the interaction between airbag and the head of dummy model plays a critical role in protecting driver in crash situation since it avoids direct impact between vehicle interiors and the head of dummy model when combined with the function of seatbelt. Dummy model tends to brace backwards to the seat and straighten its arms due to inertia effect at the end of collision process.

**Fig 5:**
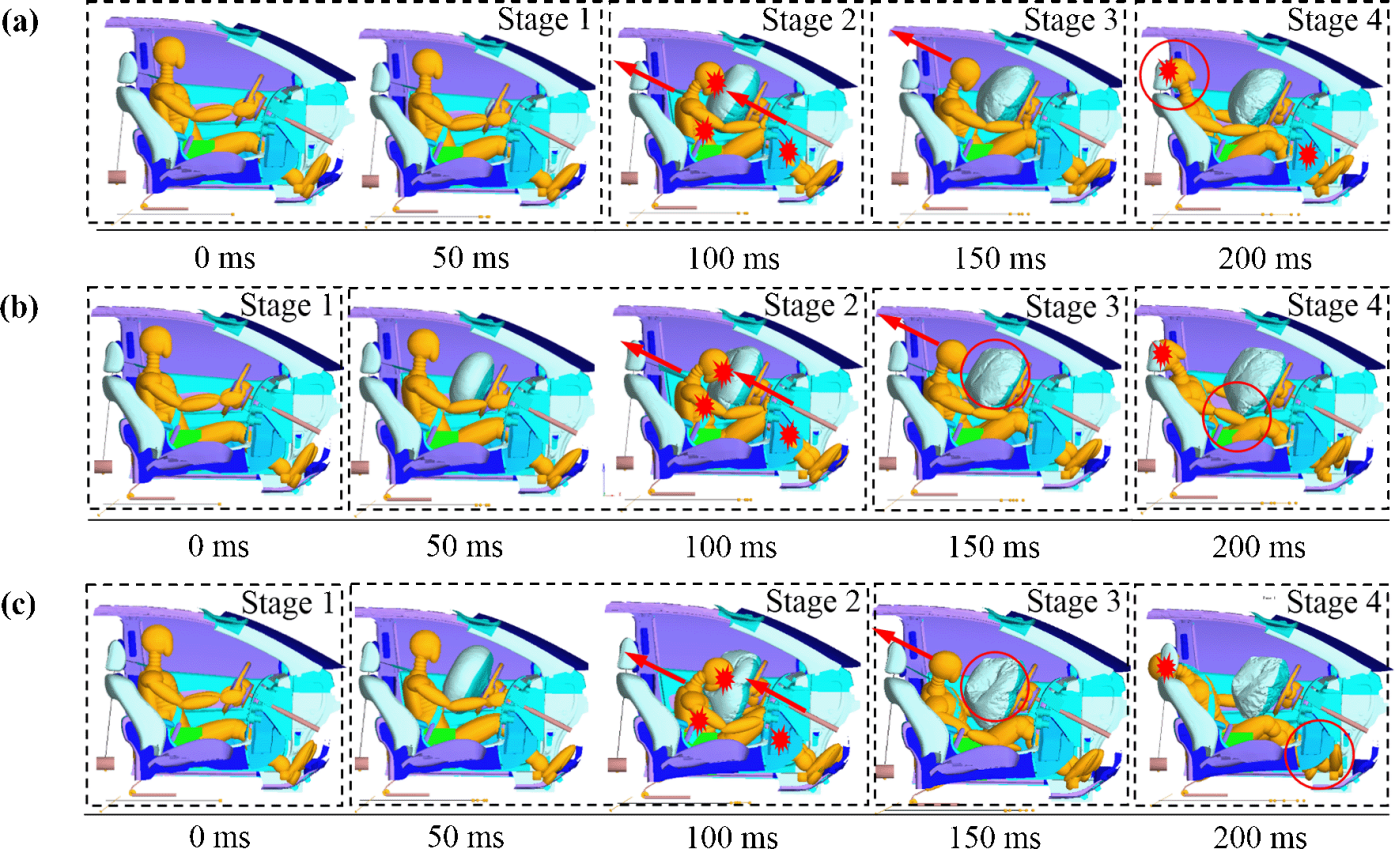
The detailed kinematic response process of drivers for the case of three different impact velocities with an impact angle of 90^o^ and collision position of position 2: (a) 25 mph, (b) 35 mph, (c) 45 mph.

In view of the consequences of drivers’ spontaneous behaviors, Fig 5 illustrates the detailed kinematic response process of drivers for the cases of three different impact velocities, divided into four stages. Stage 1 is the initial stage of vehicle-to-vehicle crash, where no distinct response of occupant restraint system is observed. In Stage 2, the airbag deploys and seatbelt is tightened along with the interaction of head and airbag, chest and seatbelt, knee and bolster, resulting in different types of injuries. Afterwards, the driver seems to move backwards to seat and separate from airbag during Stage 3. Finally, in Stage 4, the interaction of head and headrest becomes the dominant source of injury. Notably, according to the deformation of the airbag and other parts of vehicles, the driver tends to suffer severer injuries under higher impact velocities because of the effect of inertia and the sharply decreasing interior space with intrusion of the door and other parts. In addition, the interaction between driver head and airbag (Stage 2) occurs visibly earlier under higher impact velocity compared to low impact velocity.

Similar analysis could be performed on various impact angles and collision positions. On one hand, it is found that the motion processes under different impact angles are quite different. In those cases with an impact angle lower than 90^o^, driver tends to move towards door, making interactions between driver’s head and window while interaction between driver’s torso and door becomes dominant. Nevertheless, drivers appear to move in the direction to passenger seat with smaller stiffness compared with doors and other interiors of vehicle in the cases with an impact angle higher than 90^o^, thus driver injury could be relatively lower. On the other hand, although the motion processes under four collision positions seem to be similar, the initial time and duration of interaction as well as the deformation could be quite distinct. Indeed, when impact positions are close to the front of vehicles, structural differences compared to other collision positions may result in severer injuries.

## 4. Discussion

Actually, most drivers tend to brake spontaneously under emergency although the reaction time could be quite different. This critical behavior combined with the initial states of two vehicles will finally determine the impact velocity of CVCC situation. On the other hand, the steering response of driver has a decisive role in collision positions as well as impact angles. Nevertheless, steering response is much more complicated than brake reaction.

In previous literature, the impact velocity has been generally studied as a dominant factor of occupant protection considering various collision patterns [46]. Nevertheless, the impact velocity as a single variable may not illustrate the drivers’ spontaneous behavior, complicated collision process as well as all kinds of collision patterns in detail, which is exactly the major advantage of our established methodology. Consequently, the coupling effect of impact angle, collision position and the impact velocity is comprehensively investigated.

### 4.1 Coupling effect of impact velocity and collision position

A series of vehicle-to-vehicle models varying in impact angles and collision positions are established and simulated with an impact velocity of 25 mph, 35 mph and 45 mph, crash pulse from which then served as input data to corresponding occupant restraint system model to obtain drivers’ injury data under different CVCC situations.

**Fig 6:**
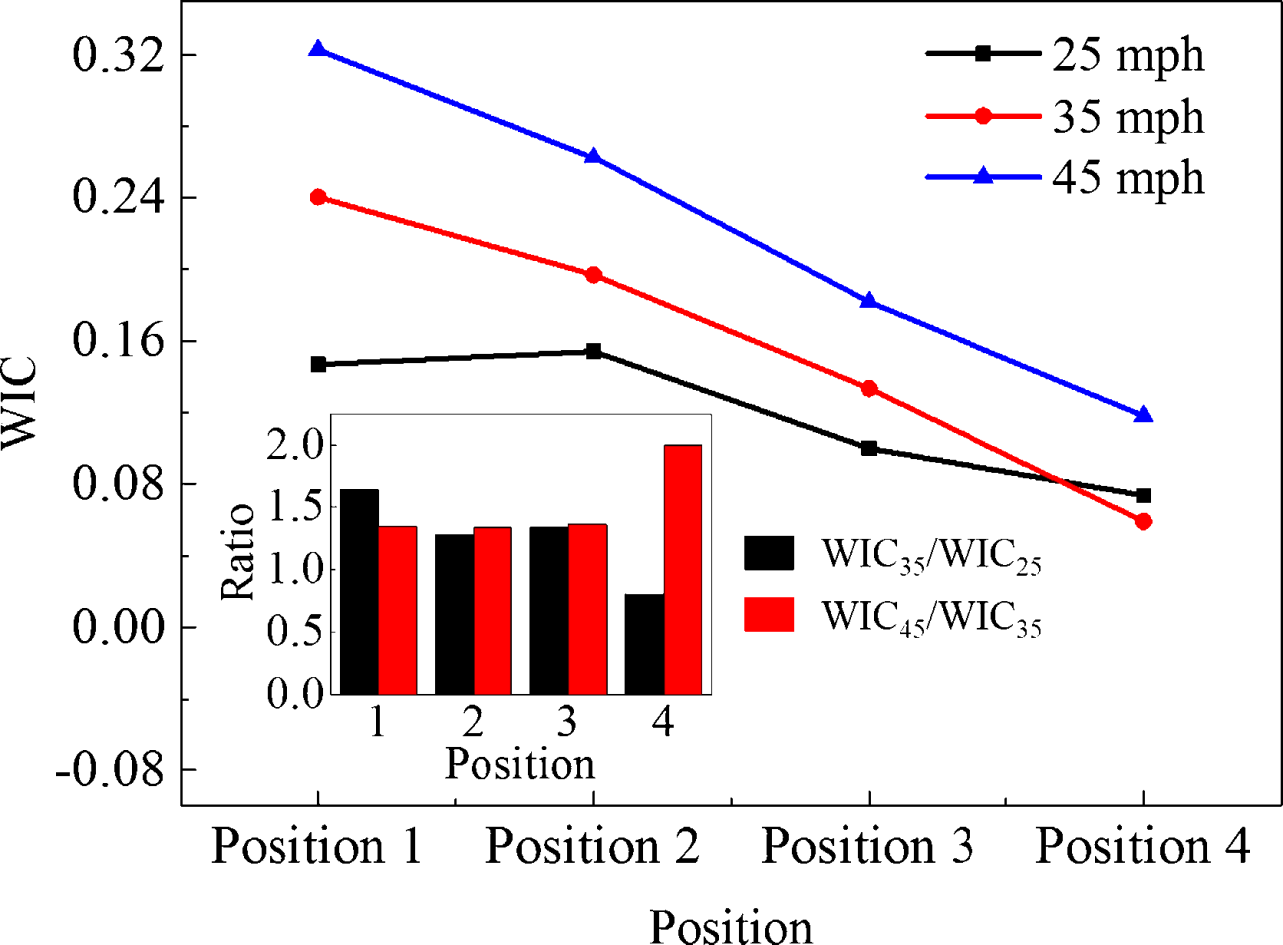
Comparison of WIC under different impact velocities and collision positions with an impact angle of 90^o^.

WIC of drivers under different impact velocities and collision positions are illustrated in Fig 6, which helps to thoroughly analyze coupling effect of impact velocity and collision position on overall injury rather than partial injury of drivers. The variation range of WIC with increasing impact velocity is 0.07 to 0.15 at 25 mph, 0.06 to 0.24 at 35 mph and 0.12 to 0.32 at 45 mph, respectively. In general, the WIC value has a positive correlation with impact velocity under different collision positions and the difference of WIC value among three impact velocities under front collision position is distinctly higher than that under rear collision position. Furthermore, the ratio of WIC value among three impact velocities in Fig 6 reveals that WIC tends to increase by 38% with an increasing impact velocity of 10 mph in average and this general rule may only be applied to position 1, position 2 and position 3. Nonetheless, the seemingly unusual situation in position 4 is quite normal since interaction time between two vehicles could be longer under an impact velocity of 25 mph even if the comparing case might equip with relatively higher impact velocity, while impact velocity will switch to a dominant role as impact velocity increases continuously.

Actually, comprehensive coupling analysis of driver’s general injury under different CVCC situations may provide several valuable guidelines for collision avoidance of autonomous driving vehicles since current algorithms have difficulty in predicting kinematics of complex collision situations. Hence, it is worth taking coupling effect of impact velocity and collision position into account, especially the distinct rule under high impact velocity.

### 4.2 Coupling effect of vehicle-to-vehicle impact angle and impact velocity

To systematically study driving behavior upon various CVCC situations, the coupling effect of vehicle-to-vehicle impact angle and impact velocity is considered in the present study using five different impact angles and three impact velocities. These two critical factors may not only affect the field of view of drivers before crashing but the collision forms under the CVCC situation.

**Fig 7:**
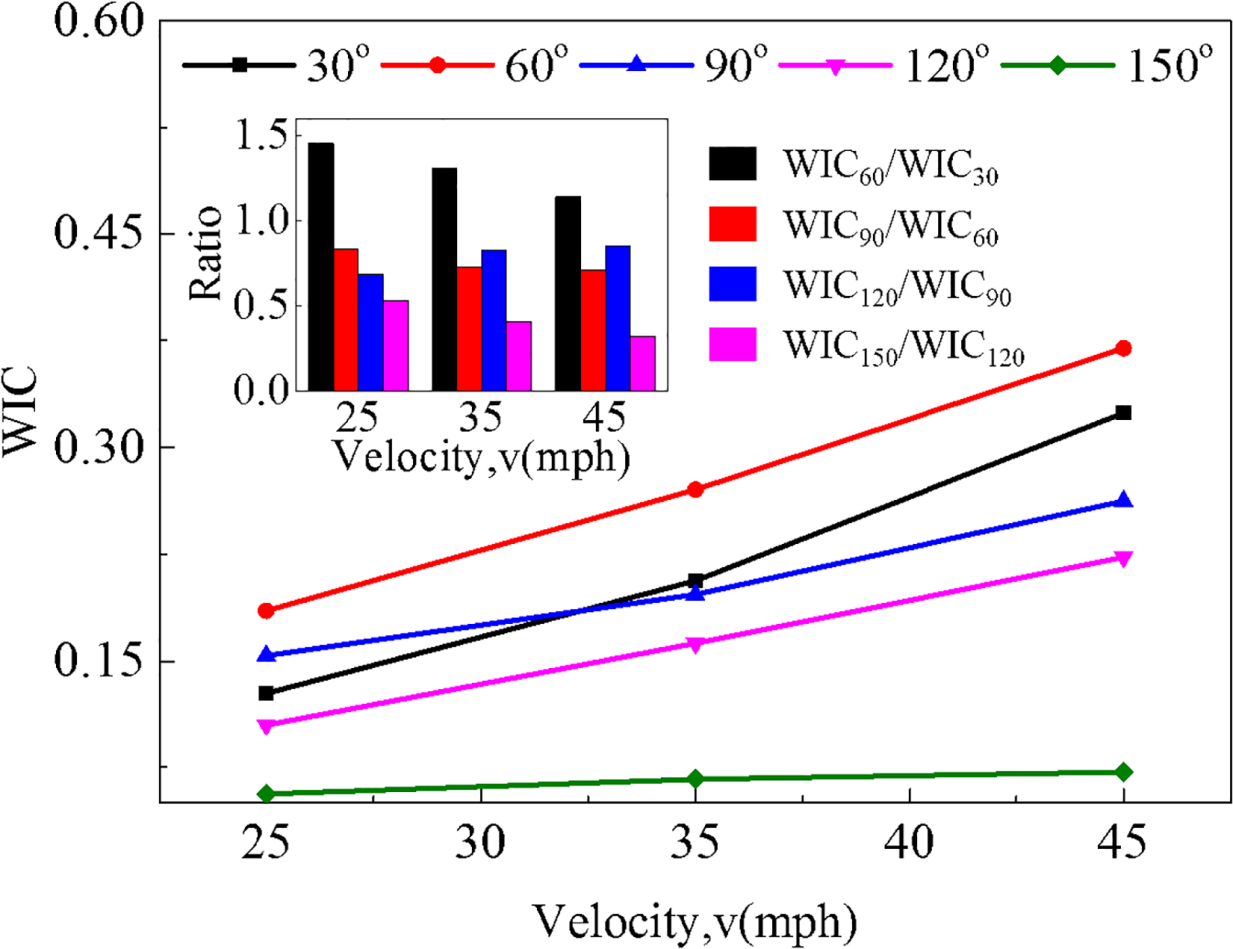
Comparison of WIC under different impact angles and impact velocities in position 2.

Fig 7 shows WIC under different impact angles and impact velocities in position 2, where the variation range of WIC value arising from various impact angles and impact velocities is 0.13 to 0.32 at 30^o^, 0.19 to 0.37 at 60^o^, 0.15 to 0.26 at 90^o^, 0.11 to 0.22 at 120^o^ and 0.06 to 0.07 at 150^o^, indicating that WIC with an impact angle of 150^o^ tends to be distinctly lower than other impact angles since vehicles separate with each other soon after collisions happen. Namely, shorter duration of interaction time during a scratch collision may lead to less dangerous situations. The maximum value of WIC appears on 60^o^ and 45 mph, exposing drivers to a more dangerous situation compared with other cases. Similarly, crash scenarios with higher impact velocities appear to be more sensitive to impact angle.

In accordance with the ratio of WIC value among five different impact angles under three impact velocities, the variation law of WIC value attributed to each impact angle shows similar trend across varying values of impact velocities, which can be divided into three sections. The impact angle ranging from 30^o^ to 60^o^ can be defined as the first section, where WIC value rises at an approximately 30% rate in average with an increasing impact angle of 30^o^. However, WIC value declines steadily in the second section with an impact angle ranging from 60^o^ to 120^o^ and decreases by 23% per 30^o^. A sharp fall (about 58%) of WIC value follows from 120^o^ to 150^o^ in the third section. Overall, impact velocity is demonstrated with dominant effect on consequences, especially in higher impact velocity situation. Indeed, improved braking maneuvers of collision avoidance for autonomous driving vehicles coupling with influencing mechanism of impact angles and impact velocities can be obtained to assist brake actuators in optimum operation by adjusting acceleration change rate within a limited range.

### 4.3 Coupling effect of collision position and impact angle

From the structural aspect, the structural interaction plays a vital role in energy absorption, which may well explain the injury severity level of drivers upon CVCC situation. The structural interaction is closely related to collision position and impact angle, thus it is essential to investigate the coupling effect of these two factors during CVCC situation.

**Fig 8:**
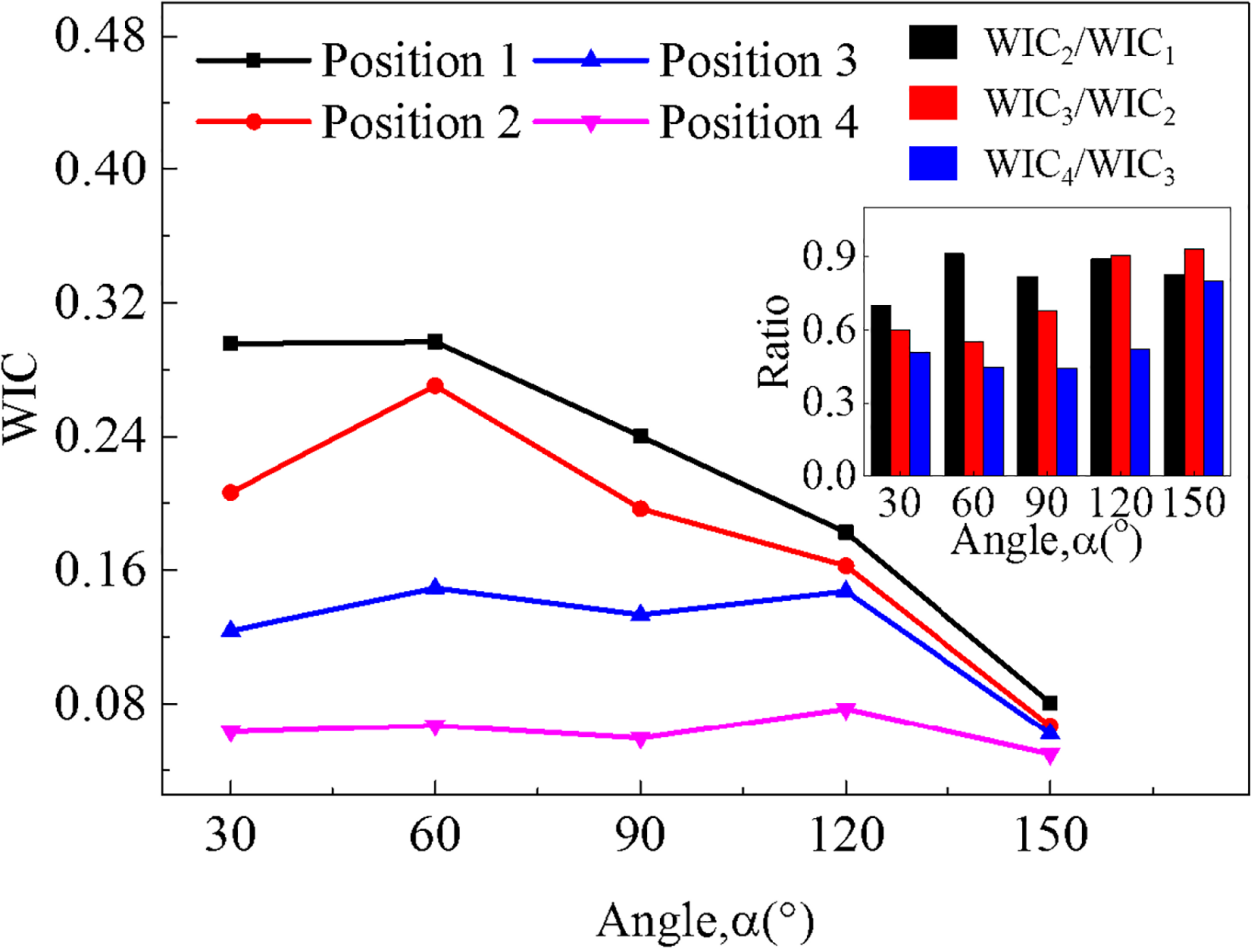
Comparison of WIC under different collision positions and impact angles with an impact velocity of 35 mph.

WICs under different collision positions and impact angles with an impact velocity of 35 mph is shown in Fig 8, which makes it clear that the general injuries of drivers tend to be severer when collision positions locate near the front of vehicles under all impact angles since actual interaction time of vehicles is longer under these circumstances. Likewise, the variation range of WIC value in accordance with an extensive parametric study is 0.08 to 0.30 at position 1, 0.07 to 0.27 at position 2, 0.06 to 0.15 at position 3, 0.05 to 0.08 at position 4, respectively, implying that injuries under position 1 appear to be several times higher than injuries under position 4.

The coupling effect can be elucidated by the fact that the difference of WIC value among four impact positions under smaller impact angles tends to be distinctly higher than that under larger impact angles. At lower impact angles (30^o^-90^o^), there is a tendency that WIC value of the rear position is 37% lower than that of the front one averagely, whereas the similar decreasing rate is only 19% at higher impact angles (120^o^-150^o^). In general, the collision position is a governing factor contributing to WIC value when compared with impact angle.

Similarly, the influencing mechanism of impact angle and collision position can help to modify controlled steering maneuvers of collision avoidance technology for autonomous driving vehicles. Under certain collision situations judged by various sensors and radar, the most reasonable steering angle and the corresponding steering angle rate combined with coordinate system can be figured out to avoid or mitigate a collision.

### 4.4 Coupling effect of impact velocity, collision position and impact angle

**Fig 9:**
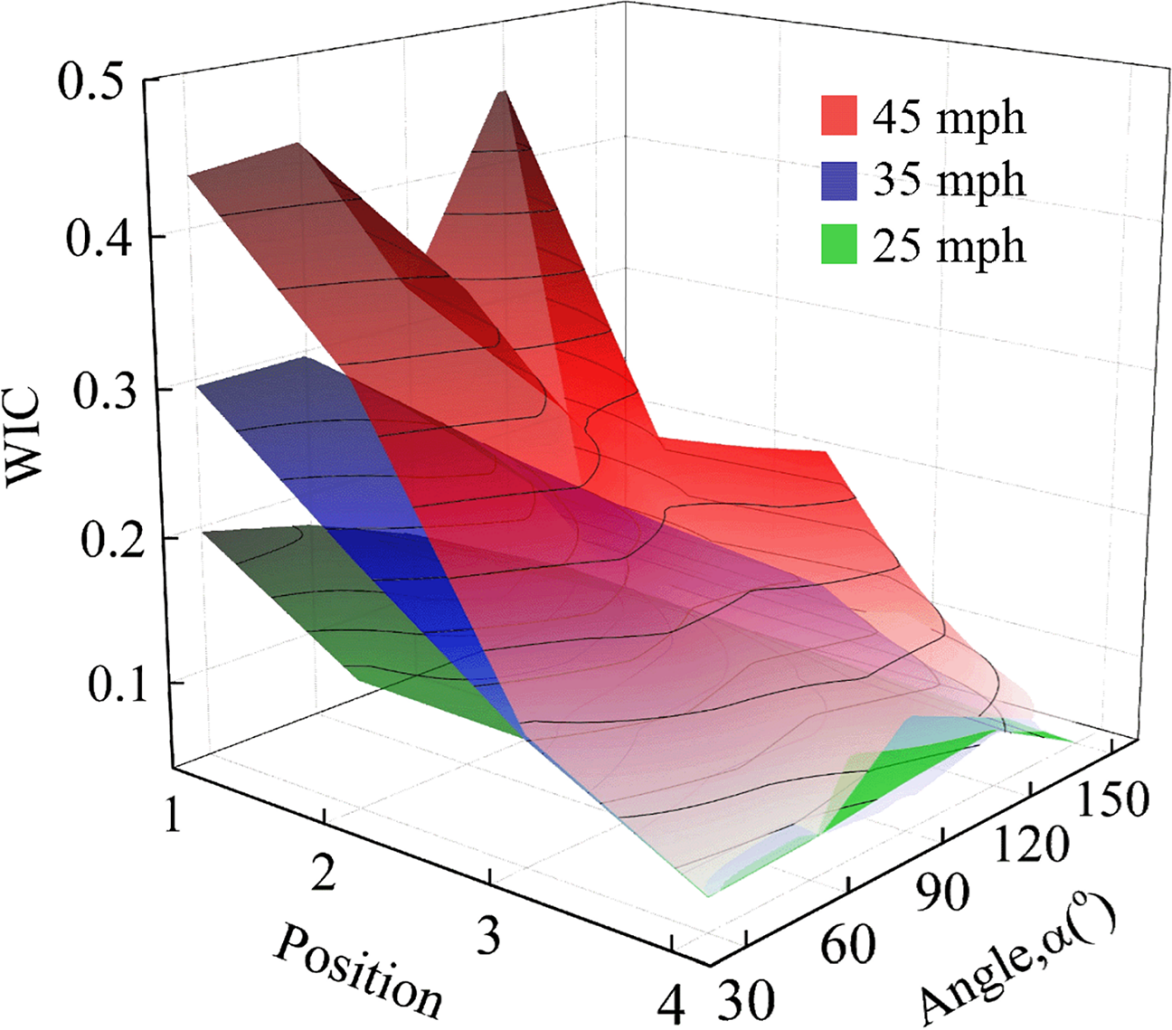
Coupling effect of the impact velocity, collision position and impact angle on WIC.

WICs resulting from various impact velocities, collision positions and impact angles are shown in Fig 9, indicating that relatively high impact velocity may enhance the sensitivity of WIC with the variation of collision positions and impact angles. For instance, WIC ranges from 0.04 to 0.20 with an impact velocity of 25 mph, 0.05 to 0.30 with an impact velocity of 35 mph and 0.06 to 0.46 with an impact velocity of 45 mph, respectively. In these cases, it is apparent that the impact velocity plays a crucial role in the value of WIC, whereas collision position serves as a secondary factor and the influence of impact angle is relatively unremarkable when compared with other two factors in general. However, for cases with impact velocity of 45 mph, the governing factor could be disparate. WIC in both 60^o^ and 120^o^ impact angle are relatively high when compared with other impact angle cases resulting from the actual impact position between driver’s body and interiors of the vehicle, demonstrating that even approximate scratch collision may lead to quite deteriorated damage under high impact velocities. Moreover, WIC value in position 3 and 150^o^ illustrates similar rule that rear collision position combined with large impact angle can also result in great risk under high impact velocities.

Actually, the distinction among the effects of three different factors on WIC is derived from diverse influencing mechanisms. In accordance with the detailed kinematic response processes of drivers in all kinds of cases, the impact velocity appears to affect the intensity of impact between dummy’s body and interiors of vehicle, resulting in increasing deformation of collision parts with the increase of impact velocity. Additionally, the impact angle has a decisive effect on the deflecting direction of driver’s body, leading to the ultimate impact position between driver’s body and interiors of the vehicle, which is also influenced by the collision position. Besides, the collision position significantly affects the direction of rotation of the vehicle. Consequently, all these factors contribute to the complicated variation of WIC in various cases, namely, the severity of driver’s general injury could be quite distinct under different CVCC situations.

## 5. Concluding remarks

Rational driver spontaneous reaction stays an essential factor to protect drivers from harm. In this study, we focus on the evaluation of drivers’ spontaneous reactions during a variety of CVCC situations based on coupled FE and MD numerical simulation. A quantitative indicator, i.e. WIC is used to describe the overall injury for the driver during an accident. Based on the numerical analysis, it could be reasonably concluded that the impact velocity has a dominant effect on the value of WIC, whereas collision position serves as a secondary factor and the influence of impact angle is relatively unremarkable by comparison. The general injury influencing mechanisms are discovered through kinematic response processes of drivers under different cases, indicating that the intensity of impact, rotating direction of vehicles as well as ultimate impact position are closely related to these factors, which will finally bring about distinct severity of injury. Nevertheless, the results under high impact velocities is quite different that large impact angles or rear positions could also result in dangerous situations.

This study provides thoroughly simulation results for crash safety of various traffic accident scenarios and serves as a preliminary step toward quantitative assessment of driver spontaneous reactions in CVCC situations, paving a new road for future safe driving training and useful insights on vehicle safety designs, as well as collision avoidance/mitigation algorithm development for autonomous driving vehicles.
